# Amelioration of Toll-like Receptor-4 Signaling and Promotion of Mitochondrial Function by Mature Silkworm Extracts in Ex Vivo and in Vitro Macrophages

**DOI:** 10.3390/nu16223932

**Published:** 2024-11-18

**Authors:** Trinh Yen Binh Phan, Byungki Jang, Sang-Kuk Kang, Jongbok Seo, Seong-Ryul Kim, Kee-Young Kim, Young Ho Koh

**Affiliations:** 1 Department of Bio-Medical Gerontology, Hallym University, Chuncheon 24252, Republic of Korea; yenbinh98chauduc@gmail.com; 2 Ilsong Institute of Life Science, Hallym University, Seoul 07247, Republic of Korea; jang@hallym.ac.kr; 3 Division of Industrial Insects and Sericulture, National Institute of Agricultural Sciences, Wanju 55365, Republic of Korea; wkdudghl@korea.kr (S.-K.K.); ksr319@korea.kr (S.-R.K.); applekky@korea.kr (K.-Y.K.); 4 Korea Basic Science Institute Seoul Center, Seoul 02841, Republic of Korea; sjb@kbsi.re.kr

**Keywords:** HongJam, supercritical fluid extracts, chronic inflammation, phagocytosis, pinocytosis

## Abstract

Objectives: The unknown immune-enhancing effects of steamed mature silkworms (*Bombyx mori* L.), known as HongJam (HJ), were investigated. Methods: Supercritical fluid extracts from the White Jade variety of HJ (WJ-SCEs) were applied to in vitro RAW264.7 macrophages (RAWMs) and ex vivo bone marrow-derived macrophages (BMDMs). Results: WJ-SCE enhanced the proliferation and viability of both RAWMs and BMDMs. Supplementation with WJ-SCE significantly reduced the lipopolysaccharide (LPS)-induced expression of iNOS mRNA and protein, resulting in decreased nitric oxide (NO) production. Additionally, WJ-SCE lowered the mRNA and protein expression of COX-2 and reduced the levels of pro-inflammatory cytokines. The mitochondrial function, ATP levels, and reactive oxygen species levels in LPS-treated macrophages were restored following WJ-SCE treatment. WJ-SCE modulated LPS-Toll-like receptor-4 (TLR-4) signaling by reducing the levels of phosphorylated (p)-p38, p-ERK1/2, and p-p65. WJ-SCE also restored gene expression related to cytokines, chemokines, glucose transport, mitochondrial metabolism, and TLR-4 signaling, suggesting the inhibition of pro-inflammatory M1 macrophage polarization. Furthermore, WJ-SCE enhanced macrophage phagocytic and pinocytotic activity. Conclusions: WJ-SCE demonstrated anti-inflammatory effects by inhibiting LPS-induced M1 polarization in both macrophage types, potentially suppressing chronic inflammation while enhancing phagocytosis and pinocytosis.

## 1. Introduction

Immunity is an absolutely essential mechanism for the survival of an organism, as it maintains homeostasis and restores normal function by eliminating foreign pathogens invading from the outside, as well as endogenous abnormality arising from mutations or damaged tissues caused by external insults. Immunity is a highly complex system involving various types of tissues and immune cells. It is broadly divided into innate immunity, which is present from birth, and acquired immunity, which develops through the infections or vaccinations encountered during an individual’s lifetime [1].

Innate immunity involves various types of immune cells, with macrophages playing crucial roles in pathogen recognition and elimination through phagocytosis, immunomodulation by regulating pro- and anti-inflammatory responses, damage detection followed by wound clearance and healing, and antigen presentation to various T-cells [1]. A key characteristic of macrophages is their wide heterogeneity and plasticity. Macrophages are activated by various stimuli, leading monocytes/macrophages to differentiate into phenotypically, functionally, metabolically, and regulatorily distinct subtypes [2].

Nonetheless, the oversimplified division of macrophages into classically activated M1 and alternatively activated M2 macrophages has been widely employed. M1 macrophages, triggered by microbial products binding to Toll-like receptors (TLRs) and cytokines, especially interferon-γ (IFN-γ), are microbicidal and pro-inflammatory. In contrast, M2 macrophages, induced by interleukin-4 (IL-4)/IL-13 and several other stimuli, play a crucial role in tissue healing, fibrosis, angiogenesis, etc [2,3]. However, any abnormality in the homeostasis and plasticity of macrophages might cause an irregular ratio of the polarized subsets of macrophages, resulting in chronic inflammation, which is one of the major causes of auto-immune diseases, arthrosclerosis, cancers, neurological disorders, and sepsis [2,4,5].

Recent studies reported that certain foods can modulate the inflammatory pathways within the body [6]. The Mediterranean and Okinawan diets have been identified as excellent examples with anti-inflammatory and -metabolic syndrome effects [7,8,9]. Based on these diets, an anti-inflammatory diet also known as “The Zone Diet” [10], the Dietary Approaches to Stop Hypertension (DASH) diet [11], and the Mediterranean with the DASH diet intervention for neurodegenerative delay (Mind) [12] have been developed and proven to reduce and delay chronic inflammation and the onset of degenerative diseases. These diets share common features, including low glycemic effects, high ratios of omega-3/omega-6 unsaturated fatty acids, no trans fatty acids, proteins from non-red meat, abundant natural phytochemicals, and vitamins [6].

HongJam (HJ) is a natural food produced by steaming and freeze-drying mature silkworm (*Bombyx mori* L.) larvae, just before they form cocoons and become pupae [13,14]. The nutritional content of HJ comprises approximately 70% proteins, 14% lipids, 3% minerals, and 1% plant-derived phytochemical compounds, including vitamins, based on dry weight [15,16,17]. Studies on the functionality of HJ have reported various health-promoting effects, including gastrointestinal protection, enhanced liver function, prevention of liver cancer metastasis, protection against Parkinson’s disease, improved memory with potential dementia prevention, skin whitening, hair growth promotion, and increased healthspan in various animal models [18,19]. These diverse effects would be difficult to achieve without substantial immune-enhancing effects, yet no study has specifically examined HJ’s immune-enhancing effects to date. Therefore, in this study, we investigated how HJ may enhance macrophage functions, which are essential in innate immunity. Due to the significant heterogeneity of in vivo macrophages, we used both in vitro and ex vivo macrophage models. To elucidate the immune-enhancing effects of WJ-SCE, we conducted biochemical, cellular, molecular, and histochemical analyses.

## 2. Materials and Methods

### 2.1. HongJam Production and Supercritical Fluid Extraction Protocols

To produce the HJ, the White Jade (WJ, also known as Baekokjam) variety of silkworm (*Bombyx mori* L.) larvae, which weave white cocoons, was used. The WJ silkworms were cultivated at the sericulture production facility of the National Institute of Agricultural Science Campus in Wanju-gun, Jeollabuk-do. Voucher samples of WJ silkworms produced in 2022 and 2023 were deposited in the Sericulture Quality Control Laboratory, Division of Industrial Insects and Sericulture.

The production of the HJ followed a previously reported method [13,14]. Briefly, mature silkworms were selected, washed, and anesthetized using tap water, followed by steaming in a pressureless steam cooker for 120 min (KumSeung, Bucheon, Republic of Korea). After steaming, the mature silkworms were rapidly frozen and freeze-dried for 48 h (Operon Ltd., Kimpo, Republic of Korea), reducing their moisture content to approximately 3%. The dried silkworms were then ground using a pulverizer and a natural stone roller (Duksan Co., Ltd., Siheung, Republic of Korea). The resulting powder was stored at −50 °C until use.

Supercritical fluid extracts of WJ-HJ (WJ-SCEs) were obtained using a Supercritical Fluid Extractor (Suflux 1L SC-CO_2_ Extraction System, ILSHIN Autoclave, Daejeon, Republic of Korea). The extraction conditions were as follows: the reservoir temperature was maintained at 22–25 °C, the extractor at 58–62 °C, the separator at 39–41 °C, the primary heater at 75 °C, and the secondary heater at 75 °C. The extraction pressure was set at 50–52 bar for the reservoir, 345–355 bar for the extractor, and 49–51 bar for the separator, with a solvent flow rate of 0.02–0.04 L/min. The extraction process was conducted for 1.5 h per 100 g of powder. Since the oils and other molecules were extracted, the remaining HJ powder was confirmed to be fully dried. The extraction efficiency was determined to be 12%.

### 2.2. Culturing Two Types of Macrophages

The study was conducted to compare the immune-enhancing effects of WJ-SCE using in vitro RAW264.7 macrophages (RAWMs) and ex vivo bone marrow-derived macrophages (BMDMs). The RAWM cells were obtained from the Korean Cell Bank and cultured in RPMI1640 completed media (Biowest, Nuaillé, France), containing 10% FBS and Penicillin/Streptomycin, under conditions of 37 °C and 5.0% CO_2_. All experiments were conducted using cells from the same passage.

The BMDMs were derived from haematopoietic stem cells isolated from the femur and tibia of 8- to 10-week-old C57BL/6J mice following the protocol published previously [20]. The animal experiments were approved and guided by the experimental animal ethics committee at Hallym University, South Korea (HMC 2022-0-1014-40). A total of 20 C57BL/6J mice were used: two mice for the cell viability and nitric oxide (NO) assays, two mice for the phagocytosis assay, one mouse for the pinocytosis assay, twelve mice for the mitochondrial I–IV activity assays, two mice for the ATP and ROS assays, and two mice for the real-time quantitative PCR (RT-qPCR) analysis. After treating the cells with RBC lysis buffer, the cells were cultured in completed DMEM (Biowest) containing 10 ng/mL Macrophage colony-stimulating factor (MCSF, Merck KGaA, Darmstadt, Germany), 10% FBS, and 1 × Penicillin/Streptomycin. After culturing for four days, media containing MCSF were added, and by day 7, most cells had differentiated into BMDMs. The differentiated cells were detached using Trypsin-EDTA and utilized for the various experiments. When treating with 100 ng/mlLPS, IFN-γ (5 ng/mL) was also applied simultaneously.

### 2.3. Cell Proliferation and Viability Assay

To investigate the proliferative effects of WJ-SCE on macrophage cells, RAWM or BMDM cells were seeded at a density of 5 × 10^4^ cells/mL in 96-well plates. After 24 h of incubation, various concentrations of lipopolysaccharide (LPS, L4381, Merck KGaA) and WJ-SCE, either individually or in combination, were applied. The proliferation of the RAWMs after another 24 h of incubation was assessed using the Quanti-Max™ WST-8 Cell Viability Assay Kit (BIOMAX, Seoul, Republic of Korea) following the manufacturer’s instructions. Briefly, after adding WST-8 solution equivalent to one-tenth of the total cell culture volume, the plates were incubated for 1 h at 37 °C and 5% CO_2_. To calculate the cell proliferation, the absorbance was measured at 450 nm and 600 nm. Since the absorbance at 600 nm reflects the turbidity of each treated group, subtracting the 600 nm absorbance from the 450 nm absorbance provides a corrected value representing the actual cell proliferation effect.

### 2.4. Nitric Oxide Detection Assay

RAWM and BMDM cells were seeded at a density of 2 × 10^5^ cells/mL in 6-well plates and incubated for 24 h. After incubation, 100 ng/mL LPS and various concentrations of WJ-SCE were applied, either individually or in combination. To measure the amount of NO produced by the cells after 48 h of incubation, the NO Plus Detection Kit (iNtRON, DaeJeon, Republic of Korea) was used. The manufacturer’s instructions were followed.

### 2.5. Macrophage Phagocytosis Assay

The protocol previously published [21] was used to investigate the effect of WJ-SCE on enhancing the phagocytic activity of RAWM and BMDM cells. The RAWM and BMDM cells (1 × 10^5^) were seeded into individual wells of an 8-well chamber slide and cultured for 24 h in media containing various concentrations of WJ-SCE. FITC-conjugated *E. coli* (K-12 strain) BioParticles (ThermoFisher Sci. Waltham, MA, USA), diluted to 1/100 in Dulbecco’s Phosphate Buffered Saline (DPBS, Biowest), were then added and incubated at 37 °C for 1 h in 5.0% CO_2_.

After incubation, the cells were fixed with 4% paraformaldehyde in 0.1M PB at room temperature for 20 min, followed by four washes with cold PBS at 10 min intervals to remove any non-phagocytosed FITC-conjugated *E. coli* BioParticles. Slides were prepared using a Vectashield mounting medium (VectorLab., Burlington, ON, Canada). Fluorescence images of the macrophages were captured using a fluorescence microscope equipped with a digital camera (KI-3000F, Korea Lab Tech, Seongnam, Republic of Korea), and the number of FITC-conjugated *E. coli* BioParticles undergoing phagocytosis was quantified.

### 2.6. Macrophage Pinocytosis Assay

The protocol previously published [21,22], a neutral red uptake assay, was used to investigate the effect of WJ-SCE on enhancing pinocytotic activity, specifically targeting low-molecular-weight solutes dissolved in the solvent in the RAWMs and BMDMs. Briefly, the RAWMs or BMDMs were seeded at a density of 5 × 10^4^ cells/mL in 96-well plates and incubated for 24 h. After various concentrations of LPS and WJ-SCE were applied, either individually or in combination, the cells were incubated for 24 h at 37 °C, 5.0% CO_2_. An equal volume of neutral red solution 0.075% (*w*/*v*) was added, and the cells were incubated for 3 h at 37 °C, 5.0% CO_2_. The remaining neutral red was washed twice with PBS. A 0.1 mL solution of cell lysate buffer (absolute ethanol and 0.02% acetic acid in a 1:1 ratio) was added to the wells, and the plates were left at room temperature overnight. The absorbance was measured at 540 nm and 600 nm. The corrected pinocytotic activity was calculated by subtracting the absorbance at 600 nm from the absorbance at 540 nm.

### 2.7. Mitochondrial Complex I–IV Activity Assays

To measure the activity of mitochondrial complexes I to IV (MitoCom I–IV) in the macrophages, the RAWMs and BMDMs were processed as previously published [23,24]. The cells were lysed using a mitochondrial extraction buffer (0.25 M sucrose, 5 mM Tris-HCl, 2 mM EGTA, and 1% BSA, pH 7.4), followed by centrifugation at 600× *g* for 5 min at 4 °C to collect the supernatant. The supernatant was then centrifuged again at 10,000× *g* to isolate the mitochondrial pellet.

The mitochondrial pellet was resuspended in 200 μL of mitochondrial extraction buffer and divided into two portions. One part (155 μL) was used for the measurement of MitoCom I–III, while the other part (45 μL) was mixed with 5 μL of 10 mM n-D-β-D maltoside to measure MitoCom IV. The activities of all MitoCom I–IV were compared against the Con group without the addition of WJ-SCE.

The activities of MitoCom I and II were measured as in previously published papers [23,24]. For the MitoCom I assay, the buffer contained 25 mM phosphate (pH 7.8), 0.35% bovine serum albumin (BSA), 60 μM dichlorophenolindophenol (DCIP), 70 μM decylubiquinone, 1 μM antimycin A, and 0.02 mM NADH (all sourced from Merck KGaA). A 20 μL sample of isolated mitochondria was combined with this buffer and 5 mM NADH, and the absorbance at 600 nm was measured for 4 min at 37 °C using a Multiskan Go spectrophotometer (Thermo Fisher Scientific, Waltham, MA, USA). For the MitoCom II assay, 20 μL of isolated mitochondria were mixed with a buffer containing 80 mM KH_2_PO4 (pH 7.8), 0.1% BSA, 2.0 mM EDTA, 0.2 mM ATP, 80 mM DCIP, 50 mM decylubiquinone, 1.0 mM antimycin A, and 3.0 mM rotenone (from Merck KGaA), and incubated for 10 min at 37 °C. Afterward, 4.0 μL of 0.1 M KCN and 2 μL of 1.0 M succinate were added to the mix, and the absorbance was measured at 600 nm for 5 min. The activities of MitoCom I and II were determined using a specific calculation formula:

Activity = (ΔA600/min × volume assay)/[(extinction coefficient of DCIP × volume of sample) × (protein concentration)],(1)

Extinction coefficient of DCIP: 19.1 mM^−1^.

The activities of MitoCom III and IV were measured as in previously published papers [23,24]. A 20 μL mitochondrial sample was combined with the MitoCom III assay buffer, which consisted of 50 mM Tris-HCl (pH 7.5), 0.1 mM decylubiquinone, 12.5 mM succinate, 2 mM potassium cyanide, 30 µM rotenone, 40 µM cytochrome c, and 4 mM NaN_3_ (all from Merck KGaA). The mixture was measured at 550 nm at 30 °C for 3 min. The enzyme activities were calculated using the following equation:

Milli OD/min/µg protein = [(ΔA550 sample − ΔA550 blank)/min]/(21.84 × volume of sample × protein concentration),(2)

For the MitoCom IV activity measurement, 0.22 mM ferrocytochrome c and 0.1 M dithiothreitol (DTT, both from Merck KGaA) were incubated at room temperature for 15 min. Afterward, 5 μL of the mitochondrial sample was added to the MitoCom IV assay buffer containing 10 mM Tris-HCl (pH 7.0), 120 mM KCl, and 11.0 µM ferrocytochrome c. The absorbance at 550 nm was recorded 7 times with 10 s intervals at 25 °C, and the enzyme activity was calculated using a specific formula:

Enzyme activity (µmol/min/mg) = (ΔA550 × volume assay)/[(21.84 × volume of sample) × (protein concentration)],(3)

21.84: ΔзmM between ferrocytochrome c and ferricytochrome c at 550 nm.

### 2.8. ATP Quantification Assay

The previously published ATP quantification assay was used [23,24]. Cells were lysed and homogenized by Phenol-TE buffer (Merck KGaA). Then, 15% of chloroform (Merck KGaA) and 10% of distilled water were added to the homogenate. The samples were vortexed and centrifuged at 10,000× *g* for 5 min at 4 °C, and then the collected supernatants were used for the ATP assay. The remaining pellets were used to determine the protein concentrations by the BCA method.

For the ATP quantification, the ATP standards and samples were mixed with ATP measurement buffer (50 μM luciferin, 1.25 μg/mL luciferase, 1 mM DTT, 20.875 mM tricine, 4.175 mM MgSO_4_, 0.835 mM EDTA, and 0.835 mM NaN_3_, Merck KGaA). The luminescence from the samples and ATP standard solutions was measured using a Victor Nivo™ multimode plate reader (PerkinElmer, Waltham, MA, USA). The amount of ATP in the samples was quantified based on the standard curve generated from the ATP standard solution, and the ATP content was normalized to the protein concentration.

### 2.9. Extracting Total RNA, cDNA Synthesis, and Real-Time Quantitative PCR Protocols

The total RNA was extracted from the macrophages using a TRIzol reagent (Thermo Fisher Sci.) as previously published [24]. The quality of the extracted total RNA was assessed by checking the A260/A280 ratio and the 28S rRNA/18S rRNA ratio. After removing the genomic DNA using DNase I, the cDNA was synthesized using the Superscript IV (Thermo Fisher Sci.).

The expression changes in the various cytokines, chemokines, TLR-4 signaling, and glucose and mitochondrial metabolism genes were analyzed using an ARIA MX RT-PCR machine and software version 1.8 (Agilent, Santa Clara, CA, USA). The expression levels of the target genes were compared and quantified using the 2^−ΔΔCT^ method. The primers and RT-q-PCR conditions used in this study are shown in Supplementary Table S1.

### 2.10. ROS Concentration Measuring Assay

After treating the macrophages with 100 ng/mL LPS and various concentrations of WJ-SCE for 24 h, either alone or in combination, the cells were harvested by centrifugation at 800× *g* for 10 min and then washed twice with ice-cold Phosphate Buffered Saline (PBS, pH 7.2). The cells were lysed using an isolation solution (PBS, pH 7.2 containing 1% Triton-X 100). After keeping the samples on ice for 10 min, the mixture was centrifuged at 13,000 rpm for 10 min. The collected supernatant was used for the ROS quantification assay.

The mixture included 50 µL of the sample, 150 µL of PBS, and 0.5 µL of 2′,7′-Dichlorofluorescin diacetate substrate (10 µM; Merck KGaA), which was incubated for 30 min at 37 °C. A multimode plate reader (Victor Nivo™, Perkin Elmer, Waltham, MA, USA) was used to measure the fluorescence absorbance of the samples at A480/535 nm (FI). The ROS levels were calculated using the following formula:

ROS level: FI/mg Protein= (ΔFI × 0.2)/(0.05)/[sample protein concentration])(4)

The results were normalized by using the activity of the inactivated macrophage group.

### 2.11. Cytokine Measurement ELISA Protocol

Pro-inflammatory macrophages activated by LPS (100 ng/mL) were treated by various concentrations of WJ-SCE for 24 h. The accumulation of Interleukin-1beta (IL-1β), Tumor Necrosis Factor-α (TNF-α), and Interleukin-6 (IL-6) in the cell culture supernatants were measured by ELISA kit (Thermo Fisher Sci.) following the user guide from the manufacturer. The results were normalized by using the Con.

### 2.12. Western Blot Analysis Protocols 

RAWMs were cultured for 24 h, and then LPS (0.5 μg/mL) and various concentrations of WJ-SCE were treated for 22 h. The cells were harvested, washed three times with cold DPBS (Biowest), and then homogenized with RIPA buffer (150 mM NaCl, 1% IGEPAL CA-630, 0.5% Na-oxycholate, 0.1% SDS, 50 mM Tris, pH 8.0; Merck KGaA) with protease and phosphatase inhibitor cocktails (Thermo Fisher Sci.). After being centrifuged at 14,000× *g* for 20 min at 4 °C, the supernatants were collected and their protein concentrations were determined by the BCA protein assay kit (Thermo Fisher Sci.). For the Western blot analysis, 40 μg of protein from each sample was separated on 12% tri-glycine polyacryl-amide SDS gel and then transferred to a nitrocellulose membrane (GE Healthcare Bio-Sciences AB, Uppsala, Sweden). The membranes were probed with goat-anti-COX-2 polyclonal antibody (1:1000, sc-1747, Santa Cruz Biotechnology, Dallas, TX, USA), rabbit-anti-iNOS (NOS2) polyclonal antibody (1:2000; sc-650, Santa Cruz Biotechnology), and mouse-anti-β-actin monoclonal antibody (1:5000; A01010, Abbkine Scientific Co., Atlanta, GA, USA).

To investigate the activation of p38, ERK, and p65, after WJ-SCE was pre-treated for 2 h, LPS (0.5 μg/mL) was incubated for the indicated time in confluent RAWMs. The cells were processed as described above for the Western blot analysis. Rabbit monoclonal anti-p-p38 (1:2000, 9215, Cell Signaling Technology, Danvers, MA, USA), p38 (1:2000; 8690, Cell Signaling Technology), p-ERK1/2 (1:2000, 4377, Cell Signaling Technology), ERK1/2 (1:2000; 4695, Cell Signaling Technology), and NF-kB p-p65 (1:1000; 3033, Cell Signaling Technology) antibodies and mouse monoclonal β-actin (1:5000; A01010, Abbkine Scientific Co.) were used. Appropriate peroxidase-conjugated secondary antibodies (ThermoFisher Sci.) and Supersignal West Pico (ThermoFisher Sci.) were used to visualize the bands. The intensities of the bands were semi-automatically quantified using a wander tool and histogram functions in the Adobe Photoshop Program (Adobe, San Jose, CA, USA), as previously published [25].

### 2.13. Statistical Analysis

Statistical analyses were performed using one-way analysis of variance followed by Tukey’s honestly significant difference post hoc test, using Microsoft Excel (Microsoft, Redmond, WA, USA). The significant differences between groups are indicated by the different letters above the error bars in the graphs, with the significance level set at *p* < 0.05.

## 3. Results

### 3.1. WJ-SCE Supplementation Increased Macrophage Proliferation and Protected Cell Viability

RAW264.7 cells (RAWMs) and bone marrow-derived macrophages (BMDMs) were used to investigate the effects of supercritical fluid extracts of White Jade (WJ)-HJ (WJ-SCE) on macrophage functions. In the RAWMs, the cell viability increased up to 2.58-fold in a dose-dependent manner with Lipopolysaccharide (LPS) treatment (Supplementary Figure S1A, F_(6, 35)_ = 39.288, *p* = 3.9 × 10^−14^). In the BMDMs, the cell viability also increased up to 1.66-fold in a dose-dependent manner with LPS treatment (Supplementary Figure S1B, F_(6, 35)_ = 44.195, *p* = 6.6 × 10^−15^).

**Figure 1:**
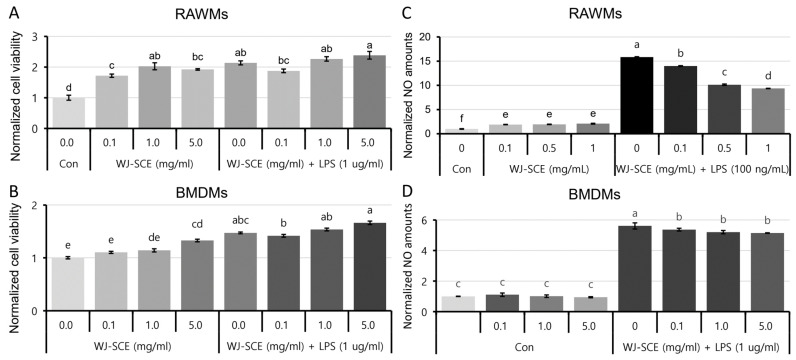
Altered cell viabilities and NO productions in RAWMs and BMDMs following treatment with various concentrations of WJ-SCE, either alone or in combination with LPS. (**A**) In RAWMs, treatment with WJ-SCE alone or in combination with LPS significantly increased cell viability (F_(7, 40)_ = 27.909, *p* = 1.7 × 10^−13^); (**B**) WJ-SCE treatment alone significantly increased cell survival, and when combined with LPS, additive effect was observed in BMDMs (F_(7, 40)_ = 27.909, *p* = 1.7 × 10^−13^); (**C**) In RAWMs, treatment with WJ-SCE alone slightly increased NO levels. LPS treatment led to 15.8-fold increase in NO levels, but when treated simultaneously with WJ-SCE, NO levels decreased in dose-dependent manner (F_(7, 40)_ = 6182.752, *p* = 0.0007); (**D**) In BMDMs, NO levels surged with LPS treatment, but co-treatment with WJ-SCE significantly reduced them (F_(7, 40)_ = 2134.3, *p* = 2.2 × 10^−49^). Different letters above error bars indicated statistically significant differences.

To investigate the effect of WJ-SCE on the macrophages, a cell viability assay was conducted (Figure 1). The groups treated with WJ-SCE alone showed a dose-dependent increase in RAWMs compared to the control group (Con). Treatment with LPS alone also significantly increased the number of RAWMs compared to the Con. However, when WJ-SCE and LPS were co-treated, there was a tendency for an increase in the cell numbers, but no significant additive effect was observed (Figure 1A, F_(7, 40)_ = 27.909, *p* = 1.7 × 10^−13^). In contrast, for the BMDMs, WJ-SCE alone showed an increase in viability at 5.0 mg/mL compared to the Con, and the group treated with both LPS and WJ-SCE displayed an additive effect (F_(7, 40)_ = 27.909, *p* = 1.7 × 10^−13^). These results indicate that WJ-SCE has protective effects that enhance the viability of macrophages.

### 3.2. WJ-SCE Reduced NO Production in Macrophages

NO is a pro-inflammatory signaling molecule secreted by macrophages in response to inflammatory stimuli like LPS. We conducted a study to examine whether WJ-SCE affected the NO production in macrophages. First, we observed the response of the macrophages to LPS treatment (Supplementary Figure S1C,D). The amount of NO produced by the RAWMs surged following LPS treatment (Supplementary Figure S1C, F_(3, 20)_ = 21,300, *p* = 3.3 × 10^−35^). Similarly, the BMDMs exhibited increased NO production in response to LPS treatment (Supplementary Figure S1D. F_(3, 20)_ = 354.09, *p* = 1.7 × 10^−17^).

We then investigated whether WJ-SCE could regulate the NO production in macrophages. When WJ-SCE was administered alone to the RAWMs, the NO production increased by 1.9 to 2.1 times compared to the Con. However, when co-treated with LPS, the increased NO levels were decreased in a dose-dependent manner by WJ-SCE (Figure 1C, F_(7, 40)_ = 6182.752, *p* = 0.0007)). In the BMDMs, there was no change in NO production following WJ-SCE treatment alone, but when co-treated with LPS, the elevated NO levels significantly decreased in all treatment groups (Figure 1D, F_(7, 40)_ = 2134.3, *p* = 2.2 × 10^−49^). These results indicate that WJ-SCE significantly reduces the LPS-induced NO production in macrophages, regardless of the type or status of the macrophages.

### 3.3. WJ-SCE Significantly Enhanced Phagocytic and Pinocytotic Activities in Macrophages

**Figure 2:**
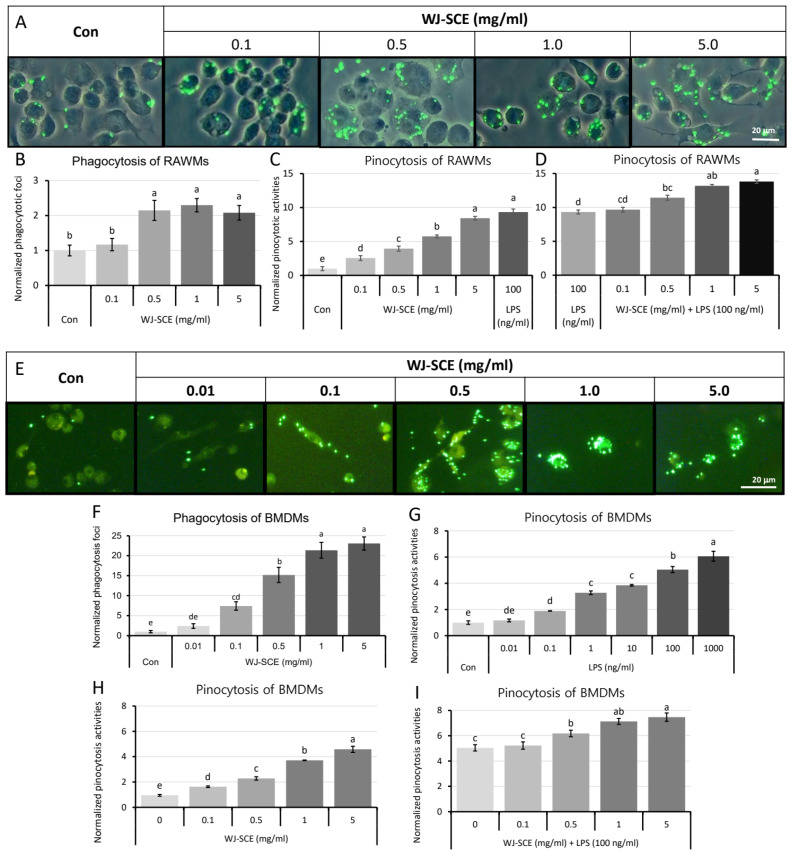
Enhanced phagocytic and pinocytotic activities in macrophages by WJ-SCE. (**A**) RAWMs treated with various concentrations of WJ-SCE showed significantly enhanced phagocytic activities with FITC-labeled *E. coli*; (**B**) phagocytic activities of RAWMs were significantly enhanced when supplemented with WJ-SCE at concentrations above 0.5 mg/mL (F_(4, 245)_ = 8.433, 2.2 × 10^−6^); (**C**) pinocytotic activities of RAWMs were significantly and dose-dependently enhanced by WJ-SCE supplementation (F_(5, 30)_ = 96.469, 1.5 × 10^−17^); (**D**) when RAWMs were co-treated with 100 ng/mL LPS and various concentrations of WJ-SCE, pinocytotic activities were significantly and dose-dependently enhanced by WJ-SCE (F_(4, 25)_ = 15.838, 1.4 × 10^−6^); (**E**) BMDMs treated with various concentrations of WJ-SCE showed significantly enhanced phagocytic activities with FITC-labeled *E. coli*; (**F**) phagocytic activities of BMDMs were significantly enhanced when supplemented with WJ-SCE at concentrations above 0.5 mg/mL (F_(5, 174)_ = 48.738, 2.3 × 10^−31^); (**G**) pinocytotic activities of BMDMs were significantly and LPS dose-dependently enhanced (F_(6, 35)_ = 88.107, 1.2 × 10^−19^); (**H**) pinocytotic activities of BMDMs were significantly and dose-dependently enhanced by WJ-SCE supplementation (F_(4, 25)_ = 110.01, 1.7 × 10^−15^); (**I**) when BMDMs were co-treated with 100 ng/mL LPS and various concentrations of WJ-SCE, pinocytotic activities were significantly and dose-dependently enhanced by WJ-SCE (F_(5, 30)_ = 53.014, 7.5 × 10^−12^). Different letters above error bars indicated statistically significant differences.

One of the most critical immune functions of macrophages is the elimination of pathogens or dead cells through phagocytosis [4]. We conducted a study to investigate whether WJ-SCE enhanced the phagocytic activity of macrophages against pathogens. First, RAWMs treated with 0.1–5.0 mg/mL of WJ-SCE were incubated with FITC-labeled *E. coli*, and the number of phagocytosed *E. coli* foci was examined. The results showed that the number of phagocytosed *E. coli* foci significantly increased in the groups treated with 0.5 mg/mL or higher concentrations of WJ-SCE compared to the Con (Figure 2A,B. F_(4, 245)_ = 8.433, 2.2 × 10^−6^).

Since another important function of macrophages is the pinocytotic uptake of lower density lipoprotein cholesterol (LDL-C) to prevent atherogenesis [5], we investigated whether WJ-SCE enhances the pinocytotic ability of RAWMs. Compared to the Con, the pinocytotic ability of the RAWMs treated with 0.1–5.0 mg/mL of WJ-SCE were dose-dependently increased, and LPS treatment also enhanced the pinocytotic ability of the RAWMs (Figure 2C. F_(5, 30)_ = 96.469, 1.5 × 10^−17^). Furthermore, when the RAWMs was co-treated with LPS and WJ-SCE, a dose-dependent increase in pinocytotic ability was also observed (Figure 2D. F_(5, 30)_ = 15.838, 1.4 × 10^−6^).

A similar experiment was conducted using BMDMs. When treated with various concentrations of WJ-SCE, there was a dose-dependent increase in the phagocytic foci of FITC-labeled *E. coli* in the BMDMs (Figure 2E,F. F_(5, 174)_ = 48.738, *p* = 2.3 × 10^−31^). Additionally, in the neutral red uptake assay to assess the pinocytotic activity, LPS treatment significantly increased the pinocytotic activity of the BMDMs in a dose-dependent manner compared to the Con (Figure 2G. F_(6, 35)_ = 88.107, *p* = 1.2 × 10^−19^). WJ-SCE treatment also led to a dose-dependent increase in pinocytotic activity in the BMDMs (Figure 2H. F_(4, 25)_ = 110.0, *p* = 1.7 × 10^−15^). To confirm the interaction between LPS and WJ-SCE in increasing pinocytotic activity, the BMDMs were co-treated with LPS and various concentrations of WJ-SCE, resulting in a significant and dose-dependent increase in pinocytotic activity (Figure 2I. F_(4, 25)_ = 53.014, *p* = 7.5 × 10^−12^).

These findings demonstrate that WJ-SCE enhances the phagocytic and pinocytotic activities of macrophages. In particular, the additive-enhanced pinocytosis observed from the LPS and WJ-SCE co-treatment groups suggested that WJ-SCE might activate signaling pathways that enhanced the pinocytosis, distinct from the LPS-TLR-4 signaling pathway.

### 3.4. WH-SCE Enhanced Mitochondrial Function in Macrophages

In our previous research, we demonstrated that the consumption of HJ enhanced mitochondrial function and increased ATP levels in mouse brain tissues [23,26]. Therefore, we conducted a study to measure the mitochondrial function and ATP levels in the macrophages after treating them with WJ-SCE, either alone or in combination with LPS.

**Figure 3:**
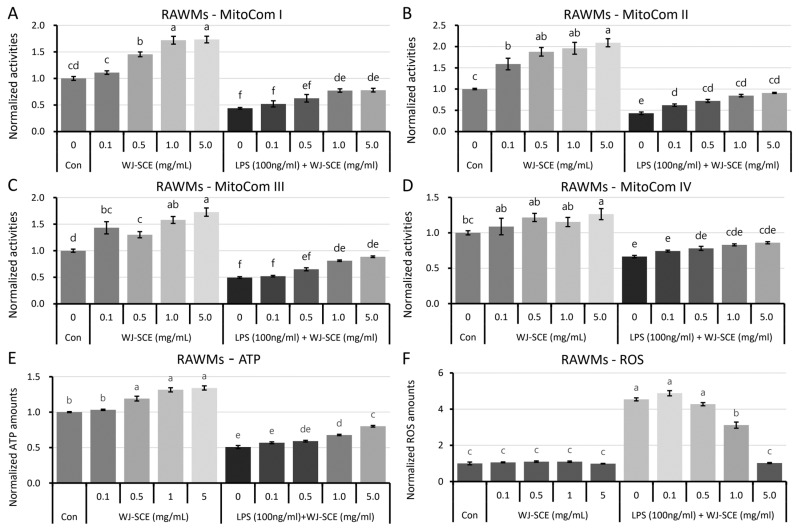
Enhanced mitochondrial function, increased ATP amounts, and reduced ROS in RAWMs by WJ-SCE supplementation; (**A**) WJ-SCE dose-dependently increased MitoCom I activity in RAWMs. Reduced MitoCom I activity caused by LPS treatment in RAWMs was restored by WJ-SCE co-treatment, making it similar to Con (F_(9, 30)_ = 91.612, *p* = 2.5 × 10^−19^); (**B**) Activity of MitoCom II in RAWMs was enhanced by WJ-SCE treatment. Reduction in MitoCom II activity caused by LPS treatment in RAWMs was restored by WJ-SCE treatment, similar to Con (F_(9, 30)_ = 61.529, *p* = 7.1 × 10^−17^); (**C**) MitoCom III activity was significantly enhanced by WJ-SCE treatment. Reduction in MitoCom III activity caused by LPS treatment in RAWMs was partially restored by WJ-SCE treatment (F_(9, 30)_ = 67.023, *p* = 2.1 × 10^−17^); (**D**) MitoCom IV activity was significantly enhanced by WJ-SCE treatment at doses greater than 1.0 mg/mL. Reduction in MitoCom IV activity caused by LPS treatment in RAWMs was partially restored by WJ-SCE treatment (F_(9, 30)_ = 22.896, *p* = 4.3 × 10^−11^); (**E**) ATP levels in RAWMs significantly increased in dose-dependent manner with WJ-SCE treatment. While reduced ATP levels in LPS-treated RAWMs were significantly increased by WJ-SCE co-treatment, they remained significantly lower than those of Con (F_(9, 30)_ = 251.64, *p* = 2.6 × 10^−32^); (**F**) ROS levels in BMDMs were not affected by WJ-SCE treatment, but significantly increased ROS levels in LPS-treated BMDMs were dose-dependently reduced by WJ-SCE, bringing them close to those of Con (F_(9, 30)_ = 383.19, *p* = 1.9 × 10^−28^). Different letters above error bars indicated statistically significant differences.

When the RAWMs were treated with various concentrations of WJ-SCE, the MitoCom I–IV activities were significantly enhanced in a dose-dependent manner (Figure 3). Additionally, the reduction in MitoCom I–IV activities (to 41.4–67.2% of the Con) in the RAWMs due to LPS treatment was significantly restored by WJ-SCE co-treatment (Figure 3A, MitoCom I: F_(9, 30)_ = 91.612, *p* = 2.5 × 10^−19^; Figure 3B, MitoCom II: F_(9, 30)_ = 61.529, *p* = 7.1 × 10^−17^; Figure 3C, F_(9, 30)_ = 67.023, *p* = 2.1 × 10^−17^; Figure 3D, F_(9, 30)_ = 22.896, *p* = 4.3 × 10^−11^). Furthermore, the ATP levels in the RAWMs treated with WJ-SCE were significantly and dose-dependently increased. While the reduced ATP levels in the LPS-treated RAWMs (50.8%) were significantly increased by WJ-SCE co-treatment, they remained significantly lower than those of the Con (Figure 3E, F_(9, 30)_ = 251.64, *p* = 2.6 × 10^−32^). The amounts of ROS in the RAWMs treated with WJ-SCE alone did not change; however, the significantly elevated ROS levels in the LPS-treated RAWMs were dose-dependently reduced by WJ-SCE to levels comparable to the Con (Figure 3F, F_(9, 30)_ = 383.19, *p* = 1.9 × 10^−28^).

**Figure 4:**
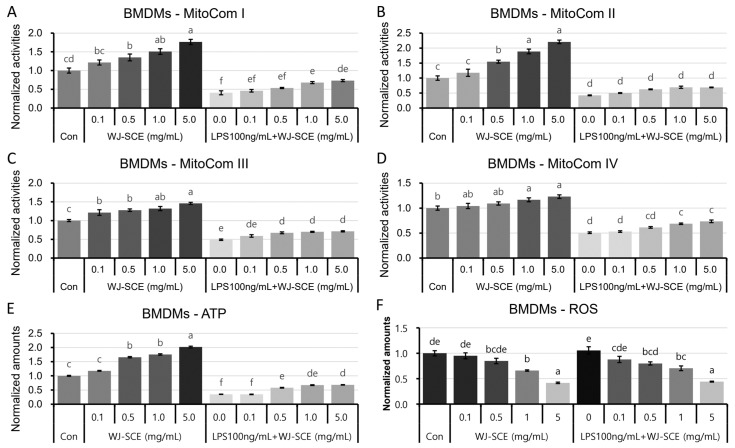
Enhanced mitochondrial function, increased ATP amounts, and reduced ROS in BMDMs by WJ-SCE supplementation; (**A**) MitoCom I activity in BMDMs significantly and dose-dependently increased with WJ-SCE treatment alone. Activity reduced by LPS treatment to 40.7% was significantly and dose-dependently increased by WJ-SCE, and in group treated with 5 mg/mL WJ-SCE, there was no statistical difference from Con (F_(9, 30)_ = 66.798, *p* = 2.2 × 10^−17^); (**B**) MitoCom II activity in BMDMs significantly and dose-dependently increased with WJ-SCE treatment alone. Activity reduced by LPS treatment to 42.2% increased with WJ-SCE treatment, but increase was not statistically significant (F_(9, 30)_ = 112.767, *p* = 1.2 × 10^−20^); (**C**) MitoCom III activity in BMDMs significantly and dose-dependently increased with WJ-SCE treatment alone. Activity reduced by LPS treatment to 48.8% significantly increased in groups treated with 0.5 mg/mL or higher of WJ-SCE, but remained lower than in Con (F_(9, 30)_ = 89.107, *p* = 3.7 × 10^−19^); (**D**) MitoCom IV activity in BMDMs significantly and dose-dependently increased with WJ-SCE treatment alone. Activity reduced by LPS treatment to 50.8% significantly increased to 73.5% in groups treated with 1.0 mg/mL or higher of WJ-SCE, but remained significantly lower than in Con (F_(9, 30)_ = 75.049, *p* = 4.3 × 10^−18^); (**E**) ATP levels in BMDMs significantly and dose-dependently increased with WJ-SCE treatment alone. Levels reduced by LPS treatment to 34.9% significantly increased to 68.2% in groups treated with 0.5 mg/mL or higher of WJ-SCE, but remained significantly lower than in Con (F_(9, 40)_ = 1213.09, *p* = 7.7 × 10^−46^); (**F**) ROS levels in BMDMs significantly and dose-dependently decreased with WJ-SCE treatment, whether administered alone or in combination with LPS (F_(9, 50)_ = 22.487, *p* = 1.2 × 10^−14^). Different letters above error bars indicated statistically significant differences.

The activities of MitoCom I–IV, as well as the ATP and ROS levels, were also assessed in the BMDMs treated with WJ-SCE alone or in combination with LPS (Figure 4). The MitoCom I–IV activities were significantly and dose-dependently enhanced by WJ-SCE treatment. Additionally, the reduced activities of MitoCom I, III, and IV due to LPS treatment in the BMDMs were significantly recovered, though still significantly lower than those of the Con (Figure 4A, MitoCom I: F_(9, 30)_ = 66.798, *p* = 2.2 × 10^−17^; Figure 4B, MitoCom III: F_(9, 30)_ = 89.107, *p* = 3.7 × 10^−19^; Figure 4D, MitoCom IV: F_(9, 30)_ = 75.049, *p* = 4.3 × 10^−18^). However, the reduction in MitoCom II activity caused by LPS treatment in the BMDMs was not recovered by WJ-SCE (Figure 4C, MitoCom II: F_(9, 30)_ = 112.767, *p* = 1.2 × 10^−20^). The ATP levels in the BMDMs were significantly and dose-dependently increased by WJ-SCE, though still significantly lower than those of the Con (Figure 4E, F_(9, 40)_ = 1213.09, *p* = 7.7 × 10^−46^). The ROS levels were significantly reduced by the WJ-SCE supplementations, regardless of LPS treatment (Figure 4F, F_(9, 50)_ = 22.487, *p* = 1.2 × 10^−14^).

### 3.5. WJ-SCE Attenuated LPS-Induced Inflammatory Cytokine Production by Macrophages

**Figure 5:**
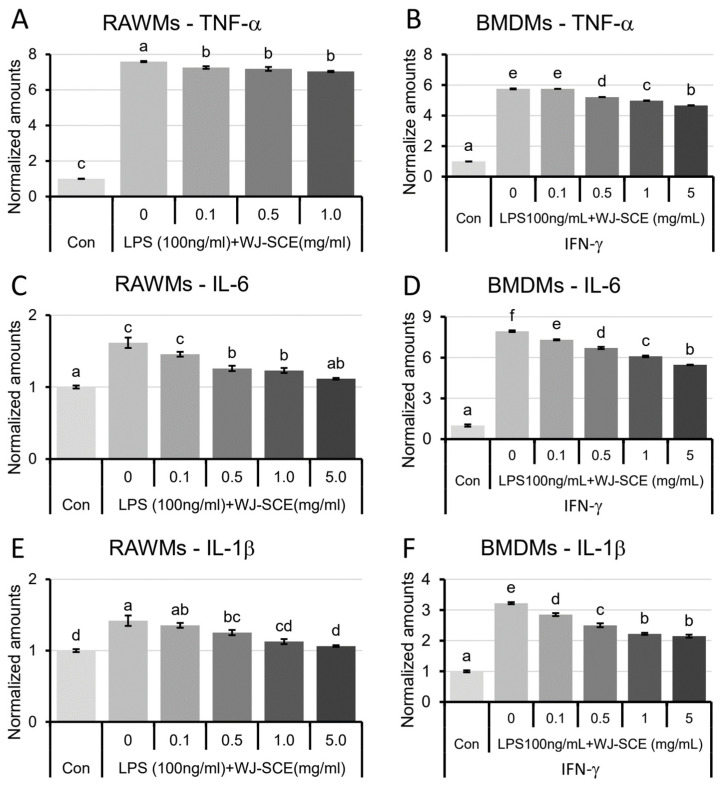
Modulation of pro-inflammatory cytokine production in macrophages by WJ-SCE supplementation. Significantly increased expressions of TNF-α ((**A**) RAWMs, F_(4, 10)_ = 2037.5, *p* = 1.7 × 10^−14^; (**B**) BMDMs, F_(5, 12)_ = 18,305.5, *p* = 7.4 × 10^−23^), IL-6 ((**C**) RAWMs, F_(5, 12)_ = 31.563, *p* = 1.7 × 10^−6^; (**D**) BMDMs, F_(5, 12)_ = 1585.6, *p* = 1.7 × 10^−16^), and IL-1β ((**E**) RAWMs, F_(5, 12)_ = 30.424, *p* = 2.0 × 10^−6^; (**F**) BMDMs, F_(5, 12)_ = 291.0, *p* = 4.4 × 10^−12^) by LPS treatment were significantly and dose-dependently reduced when WJ-SCE was co-treated. Effects of WJ-SCE were more prominent in RAWMs than BMDMs. Different letters above error bars indicated statistically significant differences.

LPS treatment in macrophages activates the TLR-4 signaling pathways, leading to the increased expression of pro-inflammatory cytokines and inducing acute inflammation for healing [4,27]. After treating RAWMs and BMDMs with LPS alone or in combination with WJ-SCE, the expression of pro-inflammatory cytokines was investigated using ELISA. The TNF-α levels, which were increased in the macrophages by LPS, were significantly reduced by WJ-SCE treatment, though it remained significantly higher than in the Con (Figure 5A. RAWM: F_(4, 10)_ = 2037.5, *p* = 1.7 × 10^−14^; Figure 5B. BMDM, F_(5, 12)_ = 18,305.5, *p* = 7.4 × 10^−23^). Similarly, IL-6, which was increased in the macrophages by LPS treatment, also showed a dose-dependent decrease with WJ-SCE supplementation. In the RAWMs, the IL-6 levels at 5.0 mg/mL of WJ-SCE were not statistically different from the Con (Figure 5C. F_(5, 12)_ = 31.563, *p* = 1.7 × 10^−6^), but in the BMDMs, the IL-6 levels decreased yet remained over four times higher than in the Con (Figure 5D, F_(5, 12)_ = 1585.6, *p* = 1.7 × 10^−16^). The IL-1β levels, which were significantly increased in the macrophages by LPS treatment, showed a dose-dependent reduction with WJ-SCE treatment in the RAWMs, and there was no statistical difference between the Con and the 1.0 mg/mL or higher WJ-SCE treatment groups (Figure 5E. F_(5, 12)_ = 30.424, *p* = 2.0 × 10^−6^). However, in the BMDMs, even with 5 mg/mL WJ-SCE treatment, the IL-1β levels were still significantly 2.15-fold higher than in the Con (Figure 5F. F_(5, 12)_ = 291.0, *p* = 4.4 × 10^−12^). These results suggest that WJ-SCE can decrease the expression of the pro-inflammatory cytokines that are increased by LPS in diverse macrophage types.

### 3.6. WJ-SCE Suppressed iNOS Expression in LPS-Stimulated Macrophages Through Inhibition of MAPK and NF-κB Signaling Cascade

**Figure 6:**
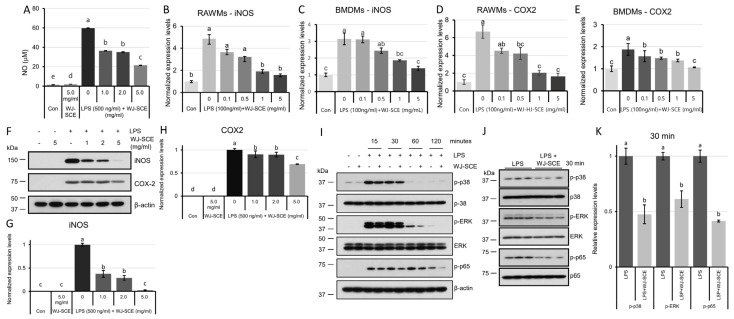
WJ-SCE reduced LPS-induced increased NO production in macrophages by reducing expression of iNOS and COX2, as well as inhibition of activation of p38, ERK, and p65; (**A**) WJ-SCE reduced LPS-induced increased NO in RAWMs (F_(5, 12)_ = 6374.9, *p* = 4.2 × 10^−20^). Expressions of iNOS mRNA in RAWMs ((**B**) F_(5, 12)_ = 29,091, *p* = 2.6 × 10^−6^) and BMDMs ((**C**) F_(5, 12)_ = 22.102, *p* = 1.1 × 10^−5^) were significant and WJ-SCE dose-dependent. Expressions of COX2 mRNA in RAWMs ((**D**) F_(5, 12)_ = 20.872, *p* = 1.5 × 10^−5^) and BMDMs ((**E**) F_(5, 12)_ = 4.065, *p* = 0.022) were significantly reduced. (**F**) Expression of iNOS and COX2 proteins was reduced by WJ-SCE supplementation in RAWMs. (**G**) Sharply increased iNOS protein expressions in RAWMs by LPS were significantly reduced in WJ-SCE-supplemented RAWMs. (**H**) Similarly, drastically increased COX2 protein expressions were significantly reduced in WJ-SCE-supplemented RAWMs. (**I**) After LPS treatments, activated p-p35, p-ERK, and p-p65 were detected. WJ-SCE co-treatment reduced expression of p-p35, p-ERK, and p-p65. (**J**,**K**) 30 min after LPS and WJ-SCE co-treatment, levels of p-p35, p-ERK, and p-65 were significantly reduced (*p* values: p-p38 = 0.00924, p-ERK = 0.00922, p-p65 = 0.00045). Different letters above error bars indicated statistically significant differences.

The excessive production of NO induced by LPS treatment in the RAWMs was significantly reduced when WJ-SCE was co-administered (Figure 6A, F_(5, 12)_ = 6374.9, *p* = 4.2 × 10^−20^). To elucidate the mechanism underlying the reduction of NO secretion by WJ-SCE in the RAWMs, we investigated whether WJ-SCE diminished the elevated expression of inducible nitric oxide synthase (iNOS) caused by LPS in the macrophages, using RT-qPCR and Western blot analysis. The expression of iNOS mRNA, which rapidly increased in the macrophages after LPS treatment, was dose-dependently reduced by WJ-SCE supplementation, reaching levels similar to those in the Con (Figure 6B. RAWMs, F_(5, 12)_ = 29,091, *p* = 2.6 × 10^−6^; Figure 6C. BMDMs, F_(5, 12)_ = 22.102, *p* = 1.1 × 10^−5^). Similarly, the expression of COX-2 mRNA, which was also upregulated by LPS in the macrophages, was dose-dependently decreased by WJ-SCE supplementation to levels that were statistically indistinguishable from the Con (Figure 6D. RAWMs, F_(5, 12)_ = 20.872, *p* = 1.5 × 10^−5^; Figure 6E. BMDMs, F_(5, 12)_ = 4.065, *p* = 0.022). The Western blot analysis of the iNOS and COX-2 protein levels in the RAWMs treated with LPS alone or in combination with WJ-SCE showed that the LPS-induced increase in iNOS was dose-dependently reduced by WJ-SCE supplementation to levels comparable to the Con (Figure 6F,G. F_(5, 12)_ = 102.05, *p* = 2.1 × 10^−9^), and the COX-2 levels were significantly reduced in all WJ-SCE-treated groups (Figure 6H,I. F_(5, 12)_ = 621.49, *p* = 4.7 × 10^−14^).

Since the activation of TLR-4 signaling by LPS leads to the phosphorylation and activation of p38, ERK1/2, and p65, which regulate the expression of iNOS and pro-inflammatory cytokines, etc. [28], we conducted a time-course study to examine the extent of the p38, ERK1/2, and p65 activation after treatment with LPS alone or in combination with WJ-SCE. The results showed that WJ-SCE consistently reduced the phosphorylation of p38, ERK1/2, and p65 triggered by LPS-TLR-4 signaling (Figure 6I). After 30 min of co-treatment with LPS and WJ-SCE, the levels of the active forms, p-p38, p-ERK1/2, and p-p65, were significantly reduced by WJ-SCE supplementation (Figure 6J,K, *p* values: p-p38 = 0.00924, p-ERK = 0.00922, p-p65 = 0.00045). These results demonstrate that WJ-SCE modulates immune responses by reducing the activation of p38, ERK1/2, and p65 in the LPS-TLR-4 signal transduction pathways, resulting in the inhibition of the MAPKinase and NF-κB signaling cascades.

### 3.7. WJ-SCE Modulated Expression of Pro-Inflammatory Genes, Glucose Transporter, and Mitochondrial Metabolism Genes Affected by LPS

**Figure 7:**
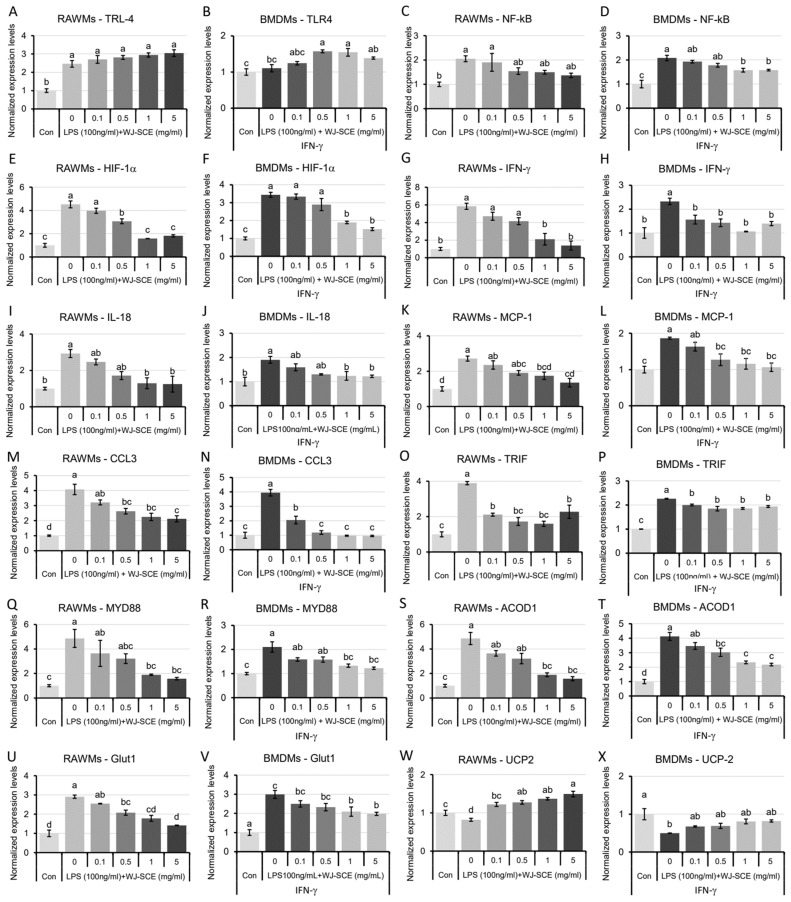
Expression changes in mRNAs of key regulators in TRL-4 signaling, pro-inflammatory responses, metabolism, and mitochondrial function induced by LPS treatments were recovered by WJ-SCE. Ex*p*ression levels of TRL-4 ((**A**) RAWMs, F_(5, 12)_ = 23.6224, *p* = 8.0 × 10^−6^; (**B**) BMDMs, F_(5, 12)_ = 23.6224, *p* = 8.0 × 10^−6^), NF-κB ((**C**) RAWMs, F_(5, 12)_ = 4.534, *p* = 0.015; 6 (**D**) BMDMs, F_(5, 12)_ = 15.833, *p* = 6.4 × 10^−5^), HIF-1α ((**E**) RAWMs, F_(5, 12)_ = 55.146, *p* = 7.3 × 10^−8^; (**F**) BMDMs, F_(5, 12)_ = 35.874, *p* = 8.2 × 10^−7^), IFN-γ ((**G**) RAWMs, F_(5, 12)_ = 19.473, *p* = 2.2 × 10^−5^; (**H**), F_(5, 12)_ = 19.473, *p* = 2.2 × 10^−5^), IL-18 ((**I**) RAWMs, F_(5, 12)_ = 8.549, *p* = 0.001; (**J**) BMDMs, F_(5, 12)_ = 5.755, *p* = 0.006), MC*p*1 ((**K**) RAWMs, F_(5, 12)_ = 11.277, *p* = 0.0003; (**L**) BMDMs, F_(5, 12)_ = 8.228, *p* = 0.0014), CCL3 ((**M**) RAWMs, F_(5, 12)_ = 22.989, *p* = 9.2 × 10^−6^; (**N**) BMDMs, F_(5, 12)_ = 45.319, *p* = 2.2 × 10^−7^), TRIF ((**O**) RAWMs, F_(5, 12)_ = 23.412, *p* = 8.4 × 10^−6^; (**P**) BMDMs, F_(5, 12)_ = 84.461, *p* = 6.3 × 10^−9^), MYD88 ((**Q**) RAWMs, F_(5, 12)_ = 6.903, *p* = 0.003; (**R**) BMDMs, F_(5, 12)_ = 12.011, *p* = 0.0002), ACOD1 ((**S**) RAWMs, F_(5, 12)_ = 10.397, *p* = 0.0005; (**T**) BMDMs, F_(5, 12)_ = 28.246, *p* = 3.1 × 10^−6^), Glut1 ((**U**) RAWMs, F_(5, 12)_ = 38.663, *p* = 5.1 × 10^−7^; (**V**) BMDMs, F_(5, 12)_ = 13.426, *p* =0.00015), and UC*p*-2 ((**W**) RAWMs, F_(5, 12)_ = 20.558, *p* = 1.7 × 10^−5^; (**X**) BMDMs, F_(5, 12)_ = 5.445, *p* = 0.008) were investigated by RT-q-*p*CR analyses. Different letters above error bars indicated statistically significant differences.

To investigate the underlying mechanisms by which WJ-SCE reduces the pro-inflammatory responses activated by LPS, we conducted an RT-q-PCR study on the expression changes of genes related to the LPS-TLR-4 signaling pathway, as well as the glucose and mitochondrial metabolisms (Figure 7). In the RAWMs, the significantly increased expression of TLR-4 mRNA due to LPS treatment did not change significantly with WJ-SCE supplementation (Figure 7A, F_(5, 12)_ = 23.6224, *p* = 8.0 × 10^−6^). In the BMDMs, while LPS treatment did not alter the expression levels of TLR-4 mRNA, a significant increase in expression was observed with WJ-SCE supplementation at concentrations of 0.5 mg/mL or higher (Figure 7B, F_(5, 12)_ = 23.6224, *p* = 8.0 × 10^−6^). Next, we compared the changes in the expression of nuclear factor kappa-light-chain-enhancer of activated B cell (NF-κB) mRNA and hypoxia-inducible factor-1 alpha (HIF-1α) mRNA, transcription factors that play crucial roles in the acute inflammation triggered by the LPS-TLR-4 signaling pathway. LPS treatment significantly increased NF-κB mRNA expression in the RAWMs and BMDMs, but this was reduced with WJ-SCE supplementation. In the 0.5 mg/mL or 1.0 mg/mL WJ-SCE treatment groups, the expression was not statistically different from the Con (Figure 7C. F_(5, 12)_ = 4.534, *p* = 0.015; Figure 7D. F_(5, 12)_ = 15.833, *p* = 6.4 × 10^−5^). Similarly, the LPS-induced increase in HIF-1α mRNA expression in the RAWMs and BMDMs was significantly reduced in the 0.5 mg/mL or 1.0 mg/mL WJ-SCE treatment groups (Figure 7E, F_(5, 12)_ = 55.146, *p* = 7.3 × 10^−8^; Figure 7F, F_(5, 12)_ = 35.874, *p* = 8.2 × 10^−7^). Interferon-gamma (IFN-γ), one of the most potent activators of macrophages, alters the macrophage metabolism to promote cell survival and maintain the pro-inflammatory state [2,29]. LPS increased the IFN-γ mRNA expression in the RAWMs and BMDMs, but with simultaneous WJ-SCE supplementation, the expression levels were not significantly different from those in the Con (Figure 7G. F_(5, 12)_ = 19.473, *p* = 2.2 × 10^−5^; Figure 7H, F_(5, 12)_ = 19.473, *p* = 2.2 × 10^−5^). Interleukin-18 (IL-18) is an effective pro-inflammatory cytokine regulating the host defense against infections, and regulates broad immune responses [30]. The significantly increased expression levels of IL-18 mRNA in the RAWMs and BMDMs treated with LPS were reduced to levels similar to those of the Con when co-treated with WJ-SCE at concentrations higher than 1.0 mg/mL (Figure 7I, F_(5, 12)_ = 8.549, *p* = 0.001; Figure 7J, F_(5, 12)_ = 5.755, *p* = 0.006).

Monocyte chemoattractant protein-1 (MCP-1) has a critical role in the process of inflammation by attracting and enhancing the expression of other inflammatory factors in immune cells [31]. The significantly increased expression of MCP-1 mRNA in the RAWMs and BMDMs by LPS were reduced to be similar to that of the Con when more than 1.0 mg/mL or 0.5 mg/mL of WJ-SCE were co-treated, respectively (Figure 7K, F_(5, 12)_ = 11.277, *p* = 0.0003; Figure 7L, F_(5, 12)_ = 8.228, *p* = 0.0014).

Chemokine (C-C motif) ligand 3 (CCL3) is a cytokine belonging to the CC chemokine family that is involved in the acute inflammatory state [32]. The LPS-induced increased expression of CCL3 mRNA in the RAWMs was significantly reduced, but still significantly higher than that of the Con (Figure 7M, F_(5, 12)_ = 22.989, *p* = 9.2 × 10^−6^). In contrast, the LPS-induced increased expression of CCL3 mRNA in the BMDMs was reduced to that of the Con (Figure 7N, F_(5, 12)_ = 45.319, *p* = 2.2 × 10^−7^).

Toll/IL-1R domain-containing adaptor-inducing IFN-β (TRIF)-dependent signaling is essential for the TLR-mediated production of type I IFN and several other pro-inflammatory mediators [28,33]. The expression levels of two out of the five TIR domain-containing adaptors were examined. The significantly increased expression of TRIF mRNA in the LPS-treated RAWMs was reduced to levels similar to those of the Con in some of the WJ-SCE-treated groups (Figure 7O, F_(5, 12)_ = 23.412, *p* = 8.4 × 10^−6^). Although the significantly increased expression of TRIF mRNA in the LPS-treated BMDMs was reduced in the WJ-SCE co-treated groups, it remained significantly higher than that of the Con (Figure 7P, F_(5, 12)_ = 84.461, *p* = 6.3 × 10^−9^). Myeloid differentiation factor 88 (MyD88) acts as the canonical adaptor for TLR signaling and the inflammation signaling pathway mediated by the interleukin-1 (IL-1) receptor family [28]. The LPS-induced sharp increases in MCP-1 mRNA expression in the RAWMs and BMDMs were dose-dependently reduced by WJ-SCE. When the macrophages were treated with 1.0 mg/mL or more of WJ-SCE, there was no significant difference compared to the Con (Figure 7Q, F_(5, 12)_ = 6.903, *p* = 0.003; Figure 7R, F_(5, 12)_ = 12.011, *p* = 0.0002).

Aconitate decarboxylase 1 (ACOD1), a mitochondrial enzyme responsible for catalyzing itaconate production, plays a context-dependent role in metabolic reprogramming, signal transduction, inflammasome regulation, and protein modification in both immune and non-immune cells [34]. The significantly increased expression of ACOD1 mRNA in the LPS-treated RAWMs and BMDMs was dose-dependently reduced by WJ-SCE. When more than 1.0 mg/mL of WJ-SCE was co-treated with the RAWMs, the ACOD1 mRNA levels were similar to those of the Con (Figure 7S. F_(5, 12)_ = 10.397, *p* = 0.0005). In the BMDMs, co-treatment with LPS and WJ-SCE at doses higher than 0.5 mg/mL significantly reduced the ACOD1 mRNA levels, though they remained significantly higher than those of the Con (Figure 7T. F_(5, 12)_ = 28.246, *p* = 3.1 × 10^−6^). Glucose transporter 1 (Glut1) is the primary rate-limiting glucose transporter in M1-like macrophages [35]. The significantly increased expression of Glut1 mRNA in the LPS-treated RAWMs and BMDMs was significantly reduced by WJ-SCE treatment, though the levels remained higher than those of the Con (Figure 7U. F_(5, 12)_ = 38.663, *p* = 5.1 × 10^−7^; Figure 7V, F_(5, 12)_ = 13.426, *p* =0.00015). Uncoupling protein 2 (Ucp2), a mitochondrial gene, regulates mitochondrial proton leakage, ATP production, and insulin secretion [36]. The significantly reduced expression of UCP2 mRNA in the LPS-treated RAWMs and BMDMs was restored by WJ-SCE co-treatment (Figure 7W, F_(5, 12)_ = 20.558, *p* = 1.7 × 10^−5^; Figure 7X, F_(5, 12)_ = 5.445, *p* = 0.008).

These gene expression analysis results suggested that the significantly altered expressions of mRNAs in the TRL-4, pro-inflammatory, and metabolism-regulating pathways by LPS in the RAWMs and BMDMs were restored by WJ-SCE supplementation. These findings indicate that WJ-SCE may exert immunomodulatory effects on acute and chronic inflammation by modulating the expressions of key pro-inflammatory cytokines, adaptors, and metabolic regulators.

## 4. Discussion

Living organisms can only extend their healthspan by ensuring that all physiological functions operate normally without disease. In previous studies, we confirmed that the healthspan of experimental animals increased following the consumption of HJ [24,37]. This improvement in healthspan is difficult to explain without an enhancement in immunity. However, until now, there have been no reports on the immune-enhancing effects of HJ. Therefore, the most significant contribution of this study is elucidating the innate immunity-boosting effects of HJ extract. Specifically, among the various types of immune cells involved in innate immunity, we investigated the immune-enhancing effects of WJ-SCE on macrophages through biochemical, cellular, histological, and molecular biological methods. To investigate the effects of WJ-SCE, we used two different types of macrophages in this study. The RAWM, an immortalized macrophage cell line derived from Balb/C mice, is frequently used in macrophage research because it can be easily cultured and proliferated. However, due to the significant heterogeneity of in vivo macrophages, the results from RAWMs cannot be immediately applied to the macrophages in living organisms [38,39]. To address this limitation, we concurrently used ex vivo BMDMs in this study, and most of our results showed nearly identical trends in both RAWMs and BMDMs. Specifically, the expression patterns of iNOS and COX-2, which are involved in the production of NO (the key mediator in inflammation), as well as the LPS-TLR-4 signaling cascade, the glucose and mitochondrial metabolism regulatory genes, and the enhanced phagocytosis of pathogens and pinocytotic activity of small solutes, were very similar in the RAWMs and BMDMs (Figure 1, Figure 2, Figure 3, Figure 4, Figure 5, Figure 6 and Figure 7). Therefore, it is predicted that WJ-SCE’s immune-boosting effects will exhibit similar trends in in vivo macrophages as well.

**Figure 8:**
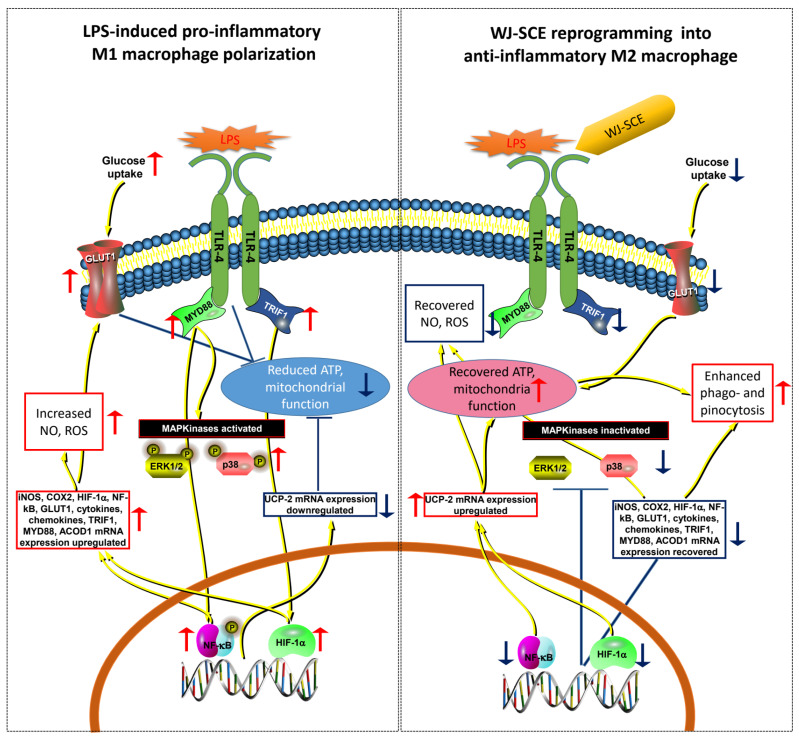
WJ-SCE reprograms LPS-induced pro-inflammatory M1 macrophages into anti-inflammatory M2 macrophages. LPS induced TLR-4 signal transduction pathway activation by phosphorylating p38, ERK1/2, and p65, resulting in upregulated expressions of iNOS, COX2, HIF-1α, NF-κB, cytokines, chemokines, TRIF1, MYD88, and ACOD1 mRNAs. NO and ROS also significantly increased, and mitochondrial function and ATP amounts were reduced in two macrophages. Treatment of WJ-SCE together with LPS restored p-p38, p-ERK1/2, and p-p65 gene expression patterns, mitochondrial function, and amounts of ATP, NO, and ROS in two macrophages. In addition, phagocytic and pinocytotic activities of macrophages were significantly enhanced. Red and blue arrows indicate upregulation and downregulation of gene expression, as well as enzyme and mitochondrial activities, respectively, while yellow-black arrows represent signal transduction pathways.

WJ-SCE significantly reduced the expression of MYD88 and TRIF1, which were elevated in the macrophages treated with LPS, either to statistically significant levels or to levels similar to those of the Con (Figure 7O–R). This result shows that WJ-SCE is involved in regulating the expression levels of at least two out of the five TIR domain-containing adaptors [29,33], inhibiting TRIF-dependent signaling by suppressing the MYD88-dependent signaling pathway and thereby reducing the phosphorylation of p38, ERK1/2, and p65, leading to a decrease in the secretion of pro-inflammatory cytokines. Simultaneously, it also inhibits the activation of the TRIF1/Interferon regulatory factor-3 cascade, reducing the production of type I interferons (Figure 7G,H and Figure 8).

Macrophages play a crucial role in the first line of defense by responding to pathogens and secreting various types of cytokines. However, under abnormal conditions, the excessive secretion of cytokines within a short period can lead to cytokine storm syndrome [40]. If the cytokine levels remain elevated for an extended period, it can result in chronic inflammation, which is a major contributor to progressive degenerative diseases [2]. In cases of cytokine storm syndrome or chronic inflammation, the excessive production of IFN-γ, IL-1β, IL-6, TNF-α, and IL-18 is a primary factor [2,40]. In this study, the simultaneous reduction in the amounts of secreted pro-inflammatory cytokines (Figure 5) and the expressions of their mRNAs (Figure 7) in both the RAWMs and BMDMs demonstrated the anti-inflammatory immunomodulation effects of WJ-SCE, which is an important finding of this research.

Another significant contribution of this study is the discovery that WJ-SCE may regulate the immune response of macrophages by enhancing mitochondrial function (Figure 3 and Figure 4). Macrophages are directly and indirectly involved in the onset and progression of various diseases. For diseases where chronic inflammation is a major contributing factor, such as cancers, atherosclerosis, and Alzheimer’s disease, it is crucial to maintain the macrophages’ mitochondrial metabolism in a state that prevents disease progression [41]. Recent studies have reported that the mitochondrial activity in immune cells is closely linked to innate immunity [42,43], and that exposure to LPS impairs the mitochondrial function in macrophages [44]. Consistent with previous research [44], this study confirmed that LPS inhibits the function of MitoCom I–IV in macrophages. WJ-SCE supplementation restored the MitoCom I–IV function in the RAWMs to levels comparable to the un-treated control, resulting in increased ATP production and reduced ROS levels (Figure 3A–D). However, in the BMDMs, while the activity of MitoCom I, III, and IV decreased by LPS treatment was recovered by WJ-SCE supplementation, it remained significantly lower than in the Con. In addition, the activity of MitoCom II was not restored by WJ-SCE supplementation (Figure 4A–D). These results are partially explained by changes in the expression of the genes involved in glucose uptake and mitochondrial metabolism, such as Glut1 and ACOD1, in the two macrophage types after WJ-SCE treatment. The overexpression of Glut1, which increases the glucose uptake in macrophages and activates the pentose phosphate pathway while reducing the cellular oxygen consumption, leading to hyper-inflammatory states [35], was reduced to the level of the Con by WJ-SCE co-treatment in the RAWMs. However, in the BMDMs, even after being reduced by WJ-SCE treatment, the Glut1 expression remained more than twice as high (Figure 7U,V). Additionally, ACOD1, which produces itaconate that accumulates in cells and inhibits mitochondrial respiration [34], was also reduced to the Con level by WJ-SCE co-treatment in the RAWMs, but in the BMDMs, the expression remained more than twice as high after WJ-SCE treatment (Figure 7S,T). The accumulated itaconate specifically inhibits succinate dehydrogenase, an enzyme crucial for the conversion of succinate to fumarate within the tricarboxylic acid cycle. This succinate accumulation inactivates MitoCom II and stabilizes HIF-1α, promoting inflammatory responses [45,46,47,48]. Consistent with these reports, the HIF-1α levels in the RAWMs were restored by WJ-SCE treatment, but remained elevated in the BMDMs even after being reduced by WJ-SCE treatment. While this study did not measure the itaconate levels in the macrophages, our findings suggest that itaconate levels may be reduced by WJ-SCE in RAWMs, but not in BMDMs. Additionally, it was confirmed that the expression levels of UCP2, which show a negative correlation with the ROS and NO levels [49], were restored by WJ-SCE treatment in both the RAWMs and BMDMs (Figure 3F, Figure 4F and Figure 7W,X). UCP2, a member of the mitochondrial anion carrier protein family, is located in the inner mitochondrial membrane. By dissipating the proton gradient across the inner mitochondrial membrane, UCP2 reduces ROS production, thereby mitigating oxidative stress. This regulation is essential for maintaining the cellular metabolism and energy balance [50,51,52,53]. Consistent with these reports, our findings suggest that WJ-SCE alters the glucose and mitochondrial metabolisms to reprogram macrophages, redirecting those polarized by LPS into the M1 pro-inflammatory state toward the anti-inflammatory M2 state (Figure 8).

Macrophages clear invading pathogens through phagocytosis and remove apoptotic dead cells via efferocytosis [54], and they remove small solutes such as LDL-C through pinocytosis, thereby preventing disease development [5,55]. Interestingly, WJ-SCE enhanced phagocytosis, which removes pathogenic microorganisms, and pinocytosis, which clears small solutes like neutral red, in both the RAWMs and BMDMs (Figure 2). This result suggests that WJ-SCE may enhance the ability of macrophages to recognize pathogen-associated molecular patterns, thereby promoting phagocytosis. The increase in pinocytosis also indicates that small solutes, including LDL-C, can be removed more efficiently, thereby boosting the immune response. The phagocytic and pinocytotic activities in macrophages are known to be directly linked to mitochondrial function and are enhanced in the anti-inflammatory M2 macrophages [55]. Therefore, the enhanced phagocytosis and pinocytosis observed in the macrophages after WJ-SCE treatment may be a result of improved mitochondrial function, which supports the polarization of the macrophages into the M2 state.

Interestingly, the freeze-dried third day of the fifth instar silkworm is known for its excellent hypoglycemic effects, and is already marketed as a health functional food in Korea. A nutritional analysis of WJ-HJ shows that it contains 0% digestible carbohydrates, meaning it has no glycemic effects. Furthermore, over 70% of its fatty acid content is unsaturated fatty acids, with more than five times as much omega-3 fatty acids compared to omega-6. Additionally, it contains abundant antioxidants, vitamins, and 39.7 times more K than Na, which helps reduce high blood pressure [56]. These nutritional characteristics strongly suggest that WJ-HJ has great potential in an anti-inflammatory diet. A clinical trial in humans will be necessary to further confirm its immune-enhancing effects.

## 5. Conclusions

In summary, WJ-SCE modulated the LPS-TLR-4 signaling in RAWMs and BMDMs by reducing the levels of NO and pro-inflammatory cytokines, with obvious anti-inflammatory effects. It also enhanced immunity by promoting the phagocytosis of pathogens and pinocytosis of other small solutes. These effects were found to be driven by the improvement in mitochondrial function through WJ-SCE supplementation. Thus, our results suggest that WJ-SCE or WJ-HJ could potentially be utilized as an anti-inflammatory dietary supplement with strong capabilities to effectively clear pathogens, dead cells, and small solutes like LDL-C, in addition to reducing the NO and pro-inflammatory cytokine production in macrophages.

## 6. Patents

A composition containing HongJam extract that enhances the proliferation and pathogen phagocytosis of macrophages; Patent Application No: 10-2023-0169351 (Republic of Korea Patent Office, 29 November 2023).

## Data Availability

The original contributions presented in this study are included in the article. Further inquiries can be directed to the corresponding author.

## Supplementary Materials

The following supporting information can be downloaded at https://www.mdpi.com/article/10.3390/nu16223932/s1. Figure S1: Altered viabilities of macrophages treated with LPS; Table S1: DNA sequences and RT-q-PCR conditions for oligomers used in this study.

## References

[1] Abbas A.K., Pillai S., Lichtman A.H. (2020). Basic Immunology: Functions and Disorders of the Immune System.

[2] Parisi L., Gini E., Baci D., Tremolati M., Fanuli M., Bassani B., Farronato G., Bruno A., Mortara L. (2018). Macrophage polarization in chronic inflammatory diseases: Killers or builders?. J. Immunol. Res..

[3] Xu H., Liew L.N., Kuo I.C., Huang C.H., Goh D.L., Chua K.Y. (2008). The modulatory effects of lipopolysaccharide-stimulated B cells on differential T-cell polarization. Immunology.

[4] Arango Duque G., Descoteaux A. (2014). Macrophage cytokines: Involvement in immunity and infectious diseases. Front. Immunol..

[5] Miyazaki T. (2022). Pinocytotic engulfment of lipoproteins by macrophages. Front. Cardiovasc. Med..

[6] Ricker M.A., Haas W.C. (2017). Anti-Inflammatory Diet in Clinical Practice: A Review. Nutr. Clin. Pract..

[7] Willcox D.C., Willcox B.J., Todoriki H., Suzuki M. (2009). The Okinawan diet: Health implications of a low-calorie, nutrient-dense, antioxidant-rich dietary pattern low in glycemic load. J. Am. Coll. Nutr..

[8] de Lorgeril M., Salen P., Martin J.L., Monjaud I., Delaye J., Mamelle N. (1999). Mediterranean diet, traditional risk factors, and the rate of cardiovascular complications after myocardial infarction: Final report of the Lyon Diet Heart Study. Circulation.

[9] Esposito K., Marfella R., Ciotola M., Di Palo C., Giugliano F., Giugliano G., D’Armiento M., D’Andrea F., Giugliano D. (2004). Effect of a mediterranean-style diet on endothelial dysfunction and markers of vascular inflammation in the metabolic syndrome: A randomized trial. JAMA.

[10] Sears B. (1995). The Zone Diet.

[11] Onwuzo C., Olukorode J.O., Omokore O.A., Odunaike O.S., Omiko R., Osaghae O.W., Sange W., Orimoloye D.A., Kristilere H.O., Addeh E. (2023). DASH diet: A review of its scientifically proven hypertension reduction and health benefits. Cureus.

[12] Kheirouri S., Alizadeh M. (2022). MIND diet and cognitive performance in older adults: A systematic review. Crit. Rev. Food Sci. Nutr..

[13] Ji S.D., Kim N.S., Lee J.Y., Kim M.J., Kweon H., Sung G., Kang P.D., Kim K.Y. (2015). Development of processing technology for edible mature silkworm. J. Seric. Entomol. Sci..

[14] Kim K.-Y., Nguyen P., Kim N.-S., Kang S.-K., Kim Y.H., Koh Y.H. (2022). New processing technology for steamed-mature silkworms (hongjam) to reduce production costs: Employing a high-speed homogenization and spray drying protocol. Korean J. Appl. Entomol..

[15] Kim Y.H., Nguyen P., Kim S.-R., Kang S.-K., Kim K.-Y., Koh Y.H. (2023). A comparison of nutritional components and memory enhancement effects of HongJam prepared from different silkworm varieties that weave yellow-colored cocoons. J. Asia-Pac. Entomol..

[16] Ji S.D., Kim N.S., Kweon H., Choi B.H., Kim K.Y., Koh Y.H. (2016). Nutrition composition differences among steamed freeze-dried mature silkworm larval powders made from 3 *Bombyx mori* varieties weaving different colored cocoons. Int. J. Indust. Entomol..

[17] Ji S.D., Kim N.S., Kweon H., Choi B.H., Yoon S.M., Kim K.Y., Koh Y.H. (2016). Nutrient compositions of *Bombyx mori* mature silkworm larval powders suggest their possible health improvement effects in humans. J. Asia-Pac. Entomol..

[18] Kim K.-Y., Koh Y.H. (2022). The past, present, and future of silkworm as a natural health food. Food Sci. Nutr..

[19] Park S.J., Kim K.-Y., Baik M.-Y., Koh Y.H. (2022). Sericulture and the edible-insect industry can help humanity survive: Insects are more than just bugs, food, or feed. Food Sci. Biotechnol..

[20] Toda G., Yamauchi T., Kadowaki T., Ueki K. (2021). Preparation and culture of bone marrow-derived macrophages from mice for functional analysis. STAR Protoc..

[21] Geng J., Shi Y., Zhang J., Yang B., Wang P., Yuan W., Zhao H., Li J., Qin F., Hong L. (2021). TLR4 signalling via Piezo1 engages and enhances the macrophage mediated host response during bacterial infection. Nat. Commun..

[22] Chen W., Zhang W., Shen W., Wang K. (2010). Effects of the acid polysaccharide fraction isolated from a cultivated *Cordyceps sinensis* on macrophages in vitro. Cell. Immunol..

[23] Nguyen P., Kim K.-Y., Kim A.Y., Kang S., Osabutey A.F., Jin H., Guo Y., Park H., Suh J.-W., Koh Y.H. (2021). The additive memory and healthspan enhancement effects by the combined treatment of mature silkworm powders and Korean angelica extracts. J. Ethnopharmacol..

[24] Mai L.X., Kang S.-K., Jo Y.-Y., Nguyen P., Kim A.-Y., Kim K.-Y., Kim N.-S., Koh Y.H. (2022). An alkaline protease-digestion of silkworm powder enhances its effects over healthspan, autophagy, and mitochondria function in a rotenone-induced Drosophila Model. Front. Nutr..

[25] Hur J.H., Lee S.-H., Kim A.Y., Koh Y.H. (2018). Regulation of synaptic architecture and synaptic vesicle pools by Nervous wreck at Drosophila Type 1b glutamatergic synapses. Exp. Mol. Med..

[26] Nguyen P., Kim K.-Y., Kim A.Y., Choi B.-H., Osabutey A.F., Park Y.H., Lee H.-T., Ji S.D., Koh Y.H. (2020). Mature silkworm powders ameliorated scopolamine-induced amnesia by enhancing mitochondrial functions in the brains of mice. J. Funct. Foods.

[27] Lu Y.C., Yeh W.C., Ohashi P.S. (2008). LPS/TLR4 signal transduction pathway. Cytokine.

[28] Kim H.-J., Kim H., Lee J.-H., Hwangbo C. (2023). Toll-like receptor 4 (TLR4): New insight immune and aging. Immun. Ageing.

[29] Wang F., Zhang S., Jeon R., Vuckovic I., Jiang X., Lerman A., Folmes C.D., Dzeja P.D., Herrmann J. (2018). Interferon gamma induces reversible metabolic reprogramming of M1 macrophages to sustain cell viability and pro-inflammatory activity. eBioMedicine.

[30] Ihim S.A., Abubakar S.D., Zian Z., Sasaki T., Saffarioun M., Maleknia S., Azizi G. (2022). Interleukin-18 cytokine in immunity, inflammation, and autoimmunity: Biological role in induction, regulation, and treatment. Front. Immunol..

[31] Singh S., Anshita D., Ravichandiran V. (2021). MCP-1: Function, regulation, and involvement in disease. Int. Immunopharmacol..

[32] Bhavsar I., Miller C.S., Al-Sabbagh M. (2015). Macrophage Inflammatory Protein-1 alpha (MIP-1 alpha)/CCL3: As abbiomarker; General methods in biomarker research and their applications. General Methods in Biomarker Research and Their Applications.

[33] Ullah M.O., Sweet M.J., Mansell A., Kellie S., Kobe B. (2016). TRIF-dependent TLR signaling, its functions in host defense and inflammation, and its potential as a therapeutic target. J. Leukoc. Biol..

[34] Wu R., Liu J., Tang D., Kang R. (2023). The dual role of ACOD1 in inflammation. J. Immunol..

[35] Freemerman A.J., Johnson A.R., Sacks G.N., Milner J.J., Kirk E.L., Troester M.A., Macintyre A.N., Goraksha-Hicks P., Rathmell J.C., Makowski L. (2014). Metabolic reprogramming of macrophages: Glucose transporter 1 (GLUT1)-mediated glucose metabolism drives a proinflammatory phenotype. J. Biol. Chem..

[36] Kim D.H., Kim H.J., Seong J.K. (2022). UCP2 KO mice exhibit ameliorated obesity and inflammation induced by high-fat diet feeding. BMB Rep..

[37] Nguyen P., Kim K.Y., Kim A.Y., Kim N.S., Kweon H., Ji S.D., Koh Y.H. (2016). Increased healthspan and resistance to Parkinson’s disease in *Drosophila* by boiled and freeze-dried mature silk worm larval powder. J. Asia-Pac. Entomol..

[38] Berghaus L.J., Moore J.N., Hurley D.J., Vandenplas M.L., Fortes B.P., Wolfert M.A., Boons G.-J. (2010). Innate immune responses of primary murine macrophage-lineage cells and RAW 264.7 cells to ligands of Toll-like receptors 2, 3, and 4. Comp. Immunol. Microbiol. Infec.t Dis..

[39] Bailey J.D., Shaw A., McNeill E., Nicol T., Diotallevi M., Chuaiphichai S., Patel J., Hale A., Channon K.M., Crabtree M.J. (2020). Isolation and culture of murine bone marrow-derived macrophages for nitric oxide and redox biology. Nitric Oxide-Biol. Chem..

[40] Fajgenbaum D.C., June C.H. (2020). Cytokine storm. N. Engl. J. Med..

[41] Missiroli S., Genovese I., Perrone M., Vezzani B., Vitto V.A.M., Giorgi C. (2020). The role of mitochondria in inflammation: From cancer to neurodegenerative disorders. J. Clin. Med..

[42] Kim S.H., Kim M.J., Lim M., Kim J., Kim H., Yun C.K., Yoo Y.J., Lee Y., Min K., Choi Y.S. (2023). Enhancement of the anticancer ability of natural killer cells through allogeneic mitochondrial transfer. Cancers.

[43] Cortés M., Brischetto A., Martinez-Campanario M.C., Ninfali C., Domínguez V., Fernández S., Celis R., Esteve-Codina A., Lozano J.J., Sidorova J. (2023). Inflammatory macrophages reprogram to immunosuppression by reducing mitochondrial translation. Nat. Commun..

[44] Ji D., Yin J.-y., Li D.-f., Zhu C.-t., Ye J.-p., Pan Y.-q. (2020). Effects of inflammatory and anti-inflammatory environments on the macrophage mitochondrial function. Sci. Rep..

[45] Langston P.K., Shibata M., Horng T. (2017). Metabolism supports macrophage activation. Front. Immunol..

[46] Wculek S.K., Dunphy G., Heras-Murillo I., Mastrangelo A., Sancho D. (2022). Metabolism of tissue macrophages in homeostasis and pathology. Cell. Mol. Immunol..

[47] Viola A., Munari F., Sánchez-Rodríguez R., Scolaro T., Castegna A. (2019). The metabolic signature of macrophage responses. Front. Immunol..

[48] Li Z., Wenbin Zheng W., Kong W., Zeng T. (2023). Itaconate: A potent macrophage immunomodulator. Inflammation.

[49] Nesci S., Rubattu S. (2024). UCP2, a member of the mitochondrial uncoupling proteins: An overview from physiological to pathological roles. Biomedicines.

[50] Mills E.L., Kelly B., Logan A., Costa A.S.H., Varma M., Bryant C.E., Tourlomousis P., Dabritz J.H.M., Gottlieb E., Latorre I. (2016). Repurposing mitochondria from ATP production to ROS generation drives a pro-inflammatory phenotype in macrophages that depends on succinate oxidation by complex II. Cell.

[51] Canton M., Rodriguez R.S., Spera I., Venegas F.C., Favia M., Viota A., Castegna A. (2021). Reactive oxygen species in macrophages: Sources and targets. Front. Immunol..

[52] Emre Y., Nubel T. (2010). Uncoupling protein UCP2: When mitochondrial activity meets immunity. FEBS Lett..

[53] Xu H., Herzel A.V., Steen K.A., Wang Q., Suttles J., Bernlohr D.A. (2015). Uncoupling lipid metabolism from inflammation through fatty acid binding protein-dependent expression of UCP2. Mol. Cell Bio..

[54] Hirayama D., Iida T., Nakase H. (2017). The phagocytic function of macrophage-enforcing innate immunity and tissue homeostasis. Int. J. Mol. Sci..

[55] Becker P.H., Thérond P., Gaignard P. (2023). Targeting mitochondrial function in macrophages: A novel treatment strategy for atherosclerotic cardiovascular disease?. Pharmacol. Therapeut..

[56] Kim N.-S., Kang S.K., Kim S.-W., Kim M.Y., Kim K.-Y., Koh Y.H. (2022). A comparative study of nutrient compositions between HongJams prepared from 5 silkworm varieties making white cocoons. Int. J. Indust. Entomol..

[57] Percie du Sert N., Hurst V., Ahluwalia A., Alam S., Avey M.T., Baker M., Browne W.J., Clark A., Cuthill I.C., Dirnagl U. (2020). The ARRIVE guidelines 2.0: Updated guidelines for reporting animal research. PLoS Biol..

