# Supplementary material for: Amelioration of Toll-like Receptor-4 Signaling and Promotion of Mitochondrial Function by Mature Silkworm Extracts in Ex Vivo and in Vitro Macrophages
Source: Nutrients. 2024 Nov 18;16(22):3932. doi: 10.3390/nu16223932 (PMC11597681; doi:10.3390/nu16223932)
Supplement: Supplementary file 1 [file nutrients-16-03932-s001.zip › nutrients-3303252-supplementary.pdf]

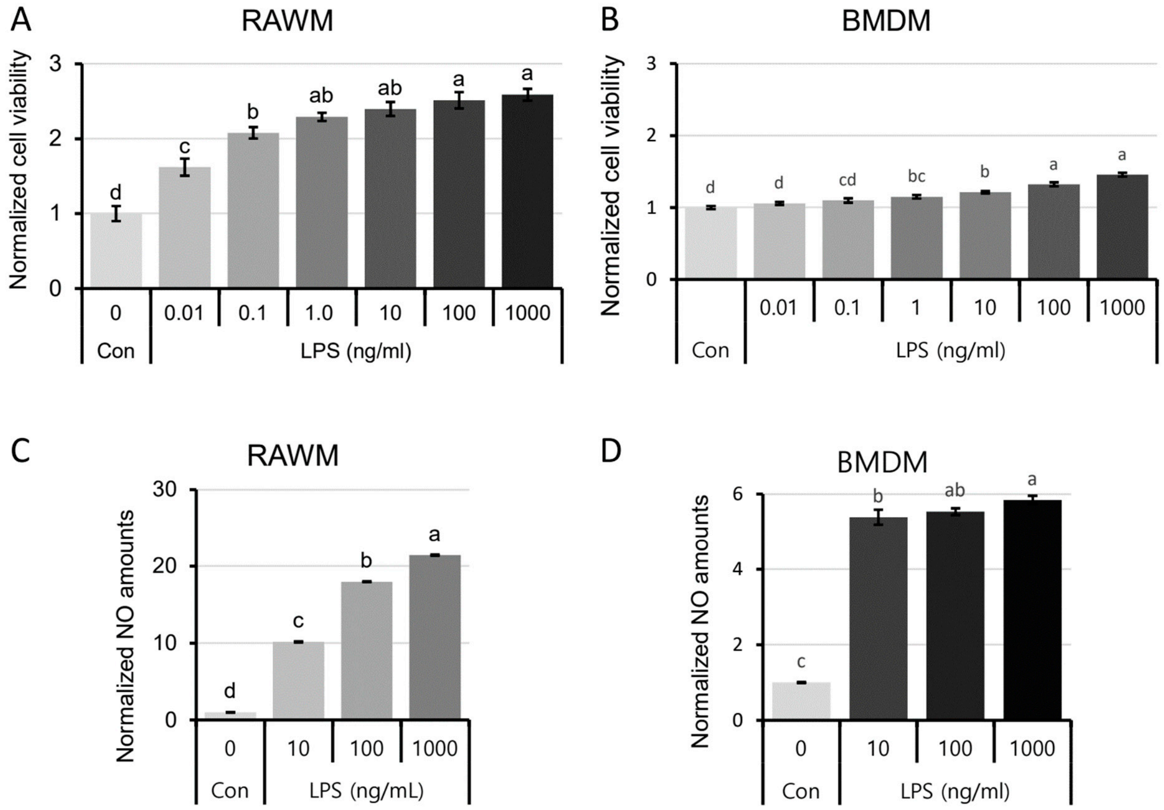

Supplementary Figure S1. Altered viabilities of macrophages treated with LPS; (A) In RAWM, the number of cells increased in a dose-dependent manner with LPS treatment ($F_{(6, 35)} = 39.288$, $P = 3.9 \times 10^{-14}$); (B) In BMDMs, a dose-dependent increase in cell survival with LPS was observed ($F_{(6, 35)} = 44.195$, $P = 6.6 \times 10^{-15}$); (C) In RAWM, the amount of NO increased sharply after LPS treatment ($F_{(4, 20)} = 21300$, $P = 3.3 \times 10^{-35}$); (D) LPS treatment significantly increased NO production in BMDM ($F_{(7, 40)} = 229.48$, $P = 3.2 \times 10^{-30}$).

Supplementary Table S1. The DNA sequences and RT-q-PCR conditions for oligomers used in this study.

Gene			DNA sequences	RT-q-PCR
Chemockines & cytokines	CCL3	F	TGCCCTTGCTGTTCTTCTCT	95 °C-2 min, 1 cycle; 95 °C-10 sec, 60 °C-15sec, 72 °C-15sec, 40 cycle
		R	GTGGAATCTTCCGGCTGTAG	
	MCP-1	F	ACTGAAGCCAGCTCTCTCTTCCTC	
		R	TTCCTTCTTGGGGTCAGCACAGAC	
	IFN-γ	F	CCAAGTTTGAGGTCAACAAC	
		R	CTTATTGGGACAATCTCTTCC	
	IL-18	F	ACTGTACAACCGCAGTAATACGG	
		R	AGTGAACATTACAGATTTATCCC	
	iNOS	F	CCTCCTCCACCCTACCAAGT	
		R	CACCCAAAGTGCTTCAGTCA	
	TNF-α	F	GTGGAACTGGCAGAAGAGGC	
		R	AGACAGAAGAGCGTGGTGGC	
	COX2	F	AGGAGACATCCTGATCCTGGT	
		R	GTTCAGCCTGGCAAGTCTTT	
TLR-4 signaling pathway	TLR4	F	TTGCTGCCAACATCATCCAG	
		R	GGTCCAAGTTGCCGTTTCTT	
	TRIF1	F	TGGCAAACACCTTCAAGACA	
		R	GCGCTTTCTTCCAGCGTA	
	MYD88	F	GCCAGAGTGGAAAGCAGTGT	
		R	CGTTGGGGCAGTAGCAGATA	
	NF-κB	F	CTGGGCACCAGTTCGATGG	

		R	GACAGCATAAGGCACACACTT	
Metabolic related genes	ACOD1	F	GCTTTTGTTAATGGTGTTGCTG	
		R	GGCTTCCGATAGAGCTGTGA	
	GLUT1		TCAACACGGCCTTCACTG	
			CACGATGCTCAGATAGGACATC	
	UCP2	F	ACTGTGCCCTTACCATGCTCC	
		R	ATTGGTAGGCAGCCATTAGGG	
	HIF-1α	F	GCCGCTGGAGACACAATCATA	95 °C-2 min, 1 cycle; 95 °C-10 sec, 52 °C-15sec, 72 °C-15sec, 40 cycle
		R	GGTGAGGGGAGCATTACATCAT	
